# Supplementary material for: A flashing light may not be that flashy: A systematic review on critical fusion frequencies
Source: PLoS One. 2022 Dec 30;17(12):e0279718. doi: 10.1371/journal.pone.0279718 (PMC9803175; doi:10.1371/journal.pone.0279718)
Supplement: S11 File — (DOCX) [file pone.0279718.s011.docx]

**A Flashing Light may not be that Flashy: a Systematic Review on Critical Fusion Frequencies**

Alix Lafitte^1,2*^, Romain Sordello^1^, Marc Legrand^1,2,3^, Virginie Nicolas^4,5^, Gaël Obein^2,6^, Yorick Reyjol^1^

**Supplementary file 11. Statistical analyses results**

**
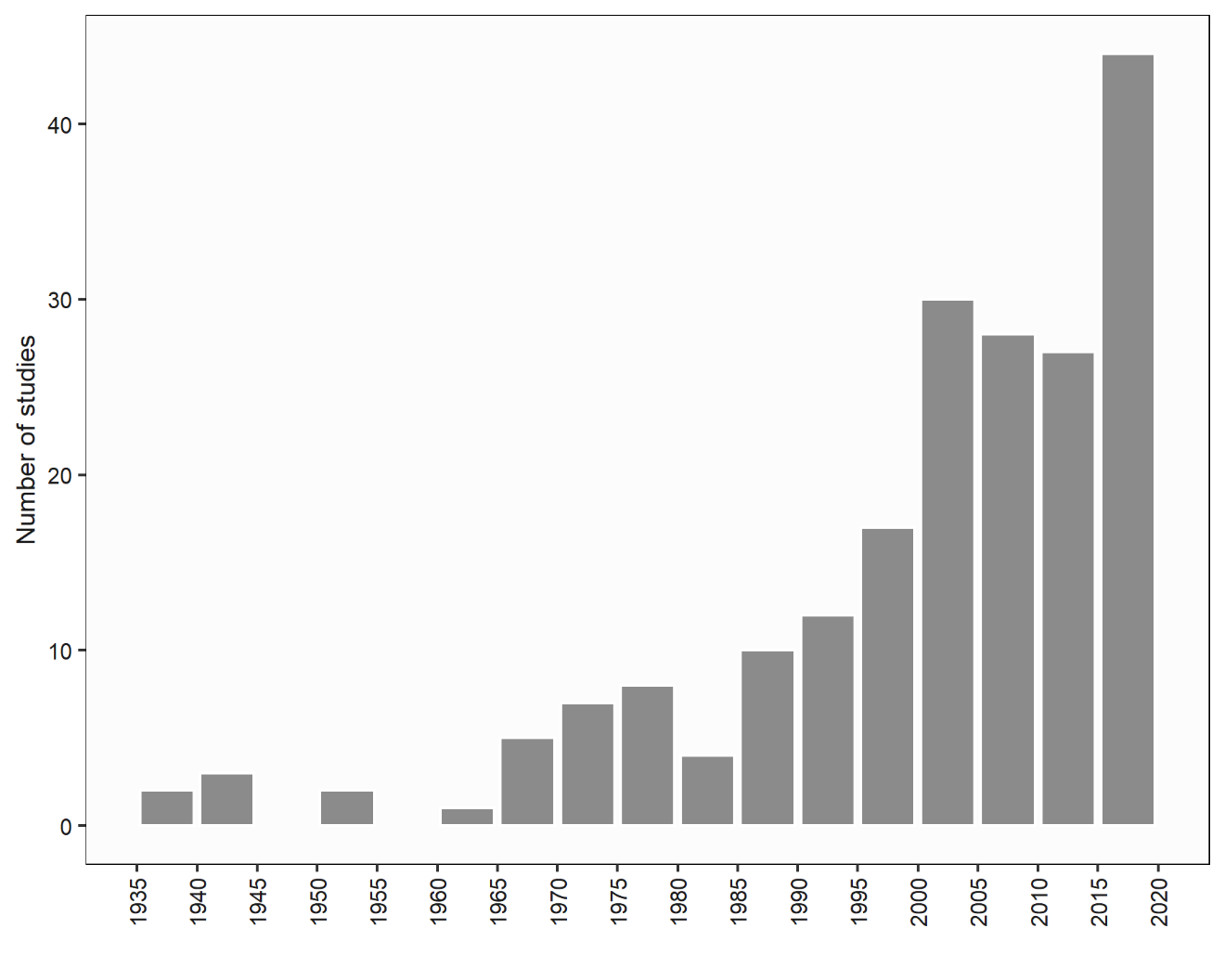
**

**S1 Fig. Number of studies by year of publication**

**
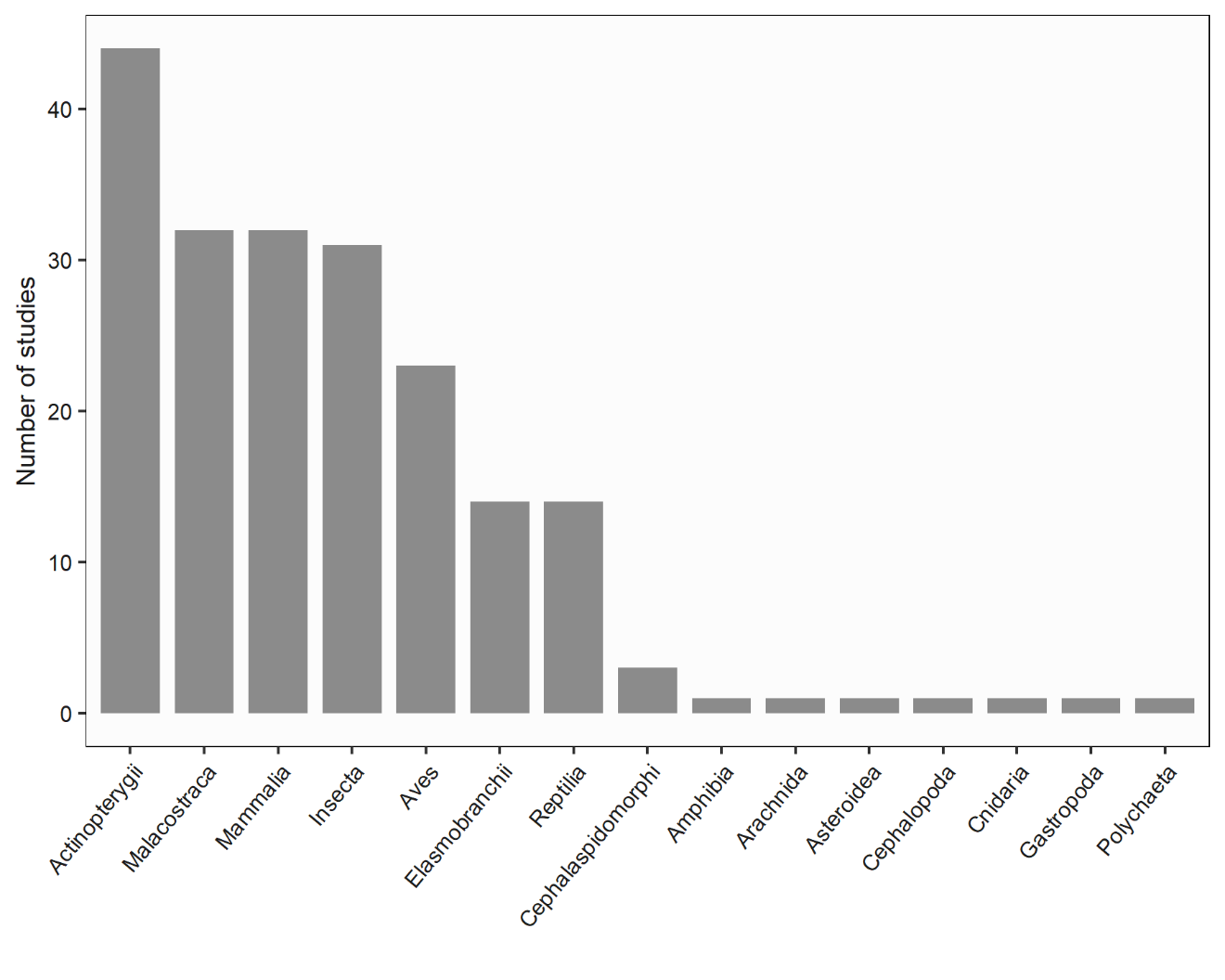
**

**S2 Fig. Number of studies by taxonomic classes**


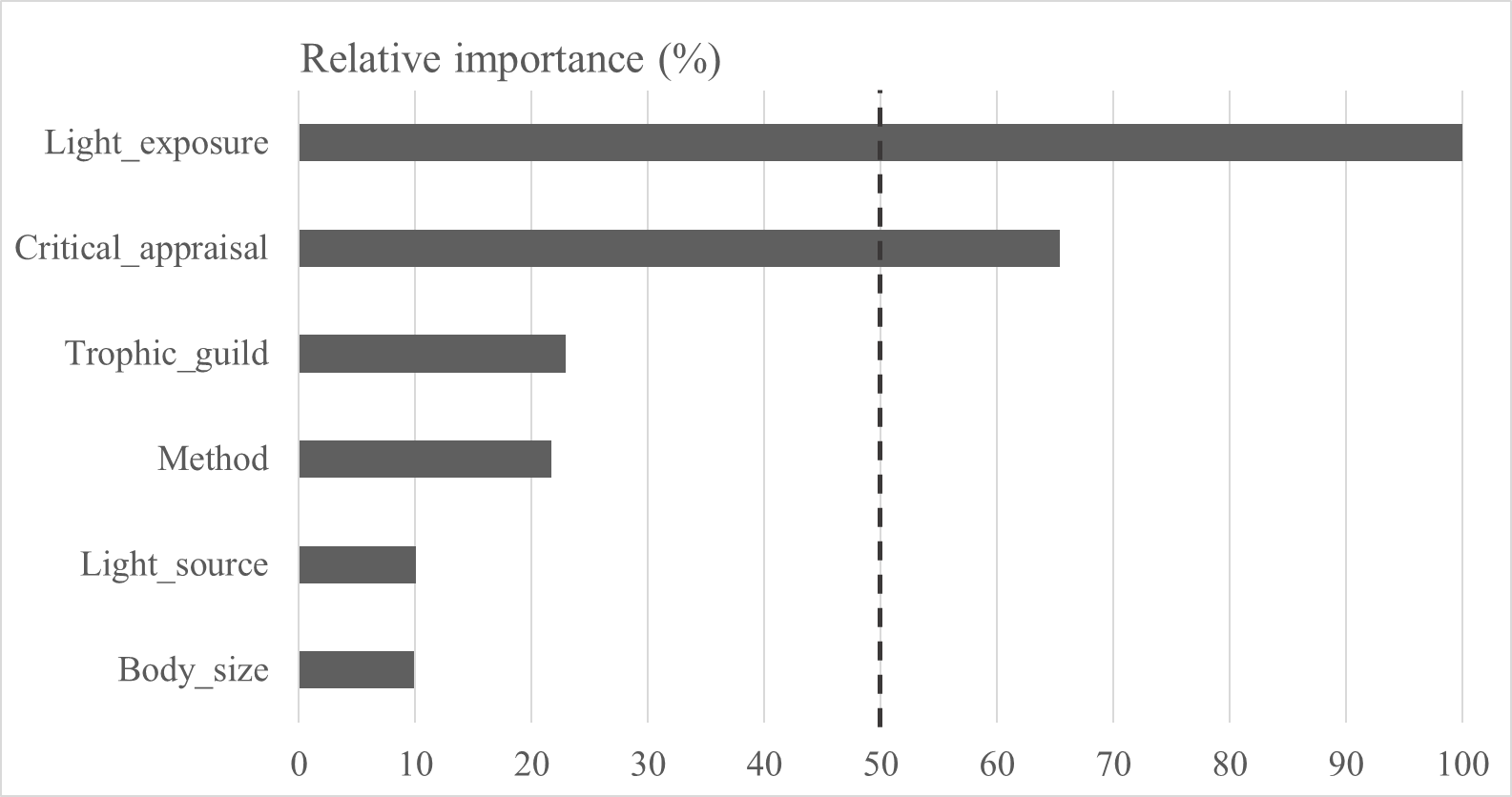


**S3 Fig.** **Relative importance of all fixed effect terms used in the second linear-mixed effect model.** Relative importance was computed as the relative cumulative Akaike weight for the set of 31 models with ΔAICc < 2—out of the 64 tested models with the dredge function provided in the R package ‘MuMIn’. Variables with a relative importance superior to 50 % were considered as relevant predictors of critical fusion frequency. Based on De Kort et al. (2021)^[[1]](#footnote-1)^

**
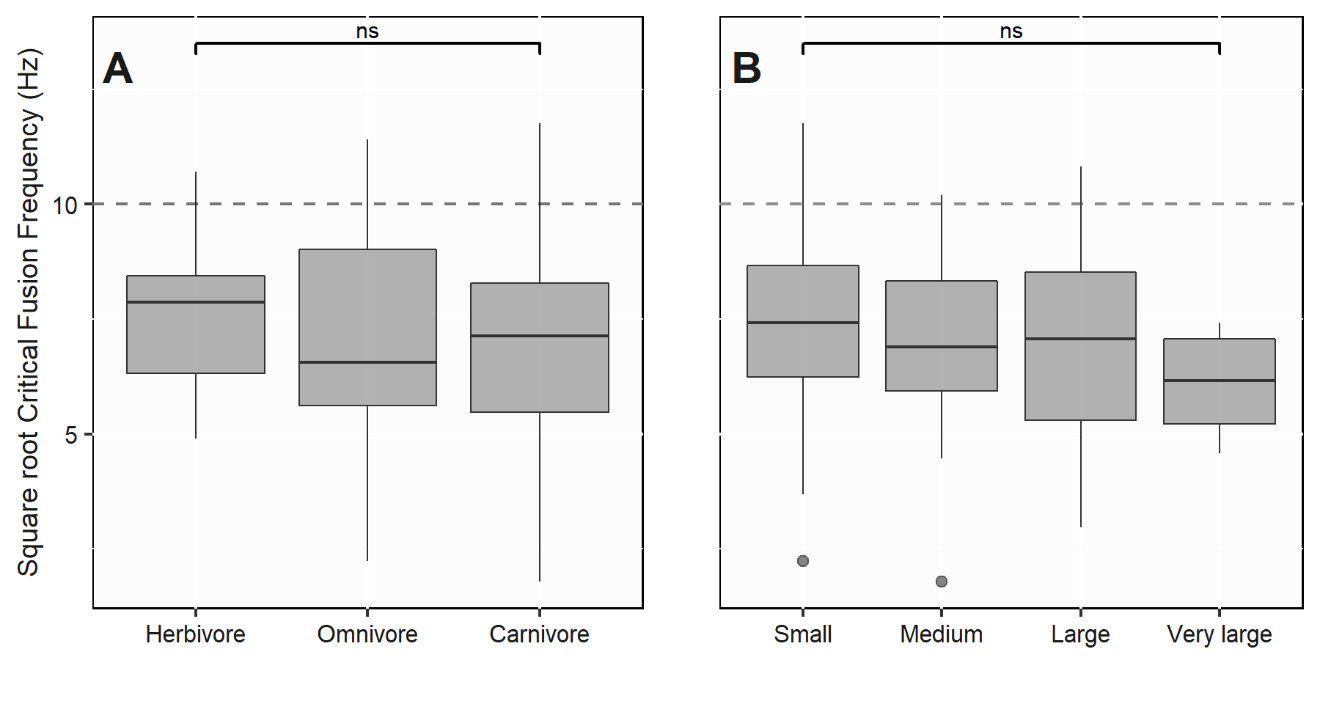
**

**S4 Fig.** **Square root Critical Fusion Frequency (CFF) for (A) herbivore, omnivore and carnivore trophic guilds and for (B) small, medium, large and very large animals.** The dashed line represents the flicker frequency of a lamp on a 50 Hz electrical supply—i.e. 100 Hz. Sample size: Herbivore (n = 12), Omnivore (n = 23), Carnivore (n = 44), Small (n = 29), Medium (n = 27), Large (n = 18), Very large (n = 5). As only one CFF value was available for animals categorised as very small, the value was discarded from the linear-mixed effect model (LMM). LMM differences are indicated as follows: *** p-value < 0.001, ** p-value < 0.01, * p-value < 0.05, ns non significant.

1. De Kort H, Prunier JG, Ducatez S, Honnay O, Baguette M, Stevens VM, et al. Life history, climate and biogeography interactively affect worldwide genetic diversity of plant and animal populations. Nat Commun. 2021;12: 516. doi:10.1038/s41467-021-20958-2 [↑](#footnote-ref-1)
