# Supplementary material for: A flashing light may not be that flashy: A systematic review on critical fusion frequencies
Source: PLoS One. 2022 Dec 30;17(12):e0279718. doi: 10.1371/journal.pone.0279718 (PMC9803175; doi:10.1371/journal.pone.0279718)
Supplement: Attachment — Submitted filename: Systematic review Critical Fusion Frequency_MINOR_COMMENTS_AE.docx [file pone.0279718.s012.docx]

**A Flashing Light may not be that Flashy: a Systematic Review on Critical Fusion Frequencies**

Alix Lafitte^1,2*^, Romain Sordello^1^, Marc Legrand^1,2,3^, Virginie Nicolas^4,5^, Gaël Obein^2,6^, Yorick Reyjol^1^

^1^ PatriNat (Office Français de la Biodiversité (OFB) – Muséum National d’Histoire Naturelle (MNHN) – Centre National de la Recherche Scientifique (CNRS)), Paris, France

^2^ Association Française de l’Eclairage (AFE), Paris, France

^3^ Université Jean Monnet, Saint-Etienne, France

^4^ Association des Concepteurs lumière et Eclairagistes (ACE), Paris, France

^5^ Concepto, Arcueil, France

^6^ Laboratoire National de métrologie et d’Essais - Conservatoire National des Arts et Métiers (LNE-CNAM), Saint-Denis, France

* Corresponding author

E-mail: alix.lafitte@mnhn.fr (AL)

# **Abstract**

**Background**: Light pollution could represent one of the main drivers behind the current biodiversity erosion. While the effects of many light components on biodiversity have been studied, the influence of flicker on animals remains poorly understood. The determination of the threshold frequency at which a flickering light is perceived as continuous by a species, usually called the Critical Fusion Frequency (CFF), could thus represent a relevant first step towards the assessment of an animal’s likelihood of being impacted by flicker.

**Objective**: This review aims at collecting CFF values for as many animal species as possible through a comprehensive, transparent and replicable systematic literature survey according to the Collaboration for Environmental Evidence standards.

**Methods**: Citations were extracted from three literature databases and were then screened for relevance successively on their titles, abstracts and full texts. Included studies were then critically appraised to assess their validity.

**Results**: At first, 4881 citations were found. Screening and critical appraisal provided 200 CFF values for 156 species. Reported values of CFF varied from a maximum of between 300 Hz and 500 Hz for the beetle *Melanophila acuminata* D. to (0.57 ± 0.08) Hz for the snail *Lissachatina fulica* B. Insects and birds had higher CFF than all other taxa studied whereas nocturnal species had lower CFF than diurnal and crepuscular ones. We also found that primary consumers might have greater CFF than species from higher levels of the food chain.

**Conclusions**: We identified nine crepuscular and nocturnal species that could be impacted by the potential adverse effects of anthropogenic light flicker. We emphasize [-or- suggest] that there remains a huge gap to fill in our knowledge of flicker perception by animals, which could potentially be hampering our understanding of its impacts on biodiversity, especially in key taxa like bats, nocturnal birds and insects.

**Keywords:** Vision, Temporal resolution, Lighting, ALAN, Dark infrastructure

# **Introduction**

We are currently facing a major crisis of biodiversity erosion [1] with species becoming extinct more and more rapidly, notably since the 1990s [2]. Simultaneously, satellite-detectable light has increased by at least 49 % between 1992 and 2017, notably due to urbanisation and Light-Emitting Diode (LED) ‘rebound effect’ [3]. Furthermore, light pollution, generated by anthropogenic light sources, has been identified as one of the main factors behind the current biodiversity erosion [4,5].

The effects of artificial light at night (ALAN) on species and ecosystems are now more and more acknowledged [6,7] and have been linked to alteration of physiology, behaviour, reproduction, habitat use, mobility and inter-species relationships [8]. In plants, light pollution can disrupt the phenology and advance the timing of budburst as shown by ffrench-Constant *et al.* (2016) [9]. At higher trophic levels, Owens *et al.* (2020) [10] reported that ALAN may alter insects’ spatial distribution and life history, thus representing one of the main anthropogenic driver behind insect decline. For birds, Dickerson, Hall & Jones (2022) [11] found that ALAN could suppress the nocturnal singing behaviour of the willie wagtail *Rhipidura leucophrys* L. and could be linked to a higher predation risk as individuals would be more easily spotted by predators under lighted conditions. Artificial light can also have adverse effects in aquatic environments and have been linked to reduced fitness in the common clownfish *Amphiprion ocellaris* C. [12]. Lastly, ALAN could also have deleterious impacts on ecosystem functioning by, for example, disrupting two key ecosystem services that are pollination and seed dispersal [13,14].

Light impacts on biological organisms have been linked to several key components of lighting, such as intensity [15–17], spectral composition [18–20], or temporality [21,22]. Flicker frequency, another component of lighting [23], can be a consequence of either the alternating nature of power supply (i.e. 50 Hz in Europe and 60 Hz in the United States) or dynamic flashing rates of anthropogenic light sources and could represent a possible additional and significant source of impacts. For instance, Greenwood *et al.* (2003) [24] found a significant behavioural preference for a continuous light compared to a flickering one in the European starling, *Sturnus vulgaris* L. Sautter, Cocchi & Schenk (2008) [25] showed that a 1.5 Hz flickering light could increase the time that rats took to get out of a water labyrinth. Barroso *et al.* (2015) [26] examined the number of captured nocturnal insects between continuous and flickering light sources and found a potential flicker avoidance effect as it lowered the number of captured Diptera, Hemiptera and Lepidoptera individuals. Flicker can also have potential significant effects on human health, as Wilkins *et al.* showed as early as 1989 [27]. The qualification and quantification of the different impacts of flickering lights is becoming increasingly important as new technologies such as LEDs now allow for more advanced dynamic lighting to appear, notably concerning shopfronts and ad panels, or the development of traffic-regulated street lamps [7,23].

Species perception of flicker may depend on their temporal resolution, which can be defined as the ability to resolve rapid movements [28]. Each species is likely to have a different temporal resolution, shaped, among other parameters, by its ecology – e.g. foraging behaviour and habitat [29,30]. A species temporal resolution can be estimated using the Flicker Fusion Frequency (FFF), defined as the threshold frequency at which a flickering light is perceived as continuous [31]. Because FFF values can vary with light intensity, Critical Fusion Frequency (CFF) is defined as the maximum flicker fusion frequency at any light intensity - i.e. the point where any further increase of light intensity does not lead to an increase of FFF [32]. A first step before assessing the impacts of flashing artificial light on animals is to build a better knowledge on CFF distribution in the animal kingdom. In this purpose, Inger *et al.* (2014) [33] published a set of 93 CFF measurements for 81 species and observed that a substantial amount of animal taxa, and especially birds and insects, were likely to perceive the flicker of a lamp on a 50 Hz or 60 Hz power supply. Healy *et al.* (2013) [34] also compiled a sample of 34 specific CFF values and showed that body mass and metabolic rate were correlated to temporal resolution. Both reviews, while useful, are not based on specific methods of evidence synthesis. The comprehensiveness or the transparency of their search strategy, screening process (inclusion/exclusion decision of all articles) or data extraction may then be limited. This could affect their robustness and lead to misinformation for decision makers, which could ultimately reduce the expected benefits of biodiversity protective actions [35,36].

Here, we propose a systematic review of animal CFF values, using the method of systematic maps and reviews as recommended by the Collaboration for Environmental Evidence [37]. Systematic reviews are based on standardized protocols that have been developed in the field of ecology over the last few years and have proven very successful to provide strong scientific evidence for practitioners [38–40]. To consolidate the existing knowledge and earlier published reviews, we used a comprehensive search strategy based on several databases and performed a critical analysis of accepted studies in order to assess their susceptibility to bias and to attribute levels of confidence in CFF values. A database was produced, containing all collected CFF values, as well as their associated metadata. This work focused on wild and domestic animals, excluding humans, and aimed at answering the following review question: until which frequency a species can perceive flicker?

# **Material & Methods**

This review, requested by professional lighting associations – i.e. the French Association on Lighting (AFE) and the French Association of Lighting Designers and Lighting Engineers (ACE) - followed the method of systemic review recommended by the Collaboration for Environmental Evidence (CEE) [37] and conformed to ROSES reporting standards [41] (S1 File). Deviations from CEE standards are listed in the section “Review limitations”.

## Search for literature

We conducted a search for literature on three accessible databases from the Web of Science platform (Clarivate): Web of Science Core Collection (WOSCC), Biological Abstracts, and Zoological Record - using the access rights provided by the French National Museum of Natural History (MNHN). These databases were selected because they cover both biology and ecology and because their functionalities enable an advanced search of literature. The WOSCC search included the following citation indexes: SCI-EXPANDED, SSCI, A&HCI, CPCI-S, CPCI-SSH, BKCI-S, BKCI-SSH, ESCI and CCR-EXPANDED. A search string was built with English terms as follows:

("fusion frequenc*"OR "flicker threshold$" OR "flicker sensitivit*" OR "flicker detection$" OR "flicker vision$" OR "flicker fusion" OR "flicker frequenc*" OR "temporal sinusoidally-modulated full-field luminance variation$")

This search string resulted from an iterative building process achieved by both ecological and physics experts from the MNHN and the National Conservatoire of Arts and Crafts (CNAM) and was used to reach the best comprehensiveness - i.e. the best recovery from a previously established test list of relevant articles. This test list included 60 articles, of which 56 came from Inger *et al.* (2014) [33] and 4 were included by the review team (S3 File). Of these 60 articles, 39 were indexed in the three databases that we used, providing an 85 % comprehensiveness (33/39). The final search was conducted on “Topic” (TS) on 1 February 2021.

## Screening process

All citations were exported and screened through a three-stage process: firstly, on titles, then, on abstracts and finally, on full-texts. Each of the three screening stages was based on predefined inclusion/exclusion criteria according to our review questions – i.e. population, exposure, outcome. Thence, we included all wild and domesticated animal species while excluding humans and other living organisms – e.g. plants, bacteria. Only artificial light sources at all wavelengths and colour temperatures were taken into account, whereas natural light sources or other types of waves like noise were excluded. We retained only citations measuring behavioural or physiological responses.

At the full-text screening stage, some additional criteria that could not be assessed at previous stages were included, regarding the language and the content and type of document. Only articles written in English and French were considered due to the review team limitations. Articles which corresponded only to an abstract were excluded, as well as reviews and studies based strictly on modelling to keep solely studies with real *in-situ* or *ex-situ* collected data. At last, articles dealing with flicker fusion which did not provide any CFF values were also discarded.

Each step of the screening process was performed by two reviewers (ML and RS), in accordance with CEE guidelines [37]. To assess the consistency of the inclusion/exclusion decisions between screeners, a Randolph’s Kappa coefficient was computed on a random set of 5 % of all articles to be screened at each step. The process was repeated until reaching a Kappa coefficient value higher than 0.6. In any case, all disagreements were discussed and resolved before beginning the screening process.

## Other sources of literature

To complete our search for literature strategy, we retrieved documents from other sources. First, we extracted references identified when carrying out critical appraisal and where CFF data were cited but were not present in our initial corpus [28,42–45]. Additionally, we included values extracted from the two main previously published reviews on CFF in our corpus [33,34]. Other relevant articles found by the review team, but not directly found in the three considered databases, were also added to the final corpus. As these articles all dealt with CFF, they did not go through any screening stage and were directly included in our corpus. A call for literature - and in particular non peer-reviewed articles published in French or in English - was also carried out by sending emails to a group of 40 experts on 12 February 2021. The corresponding documents were screened on full-texts according to the same inclusion/exclusion criteria as described above.

## Critical appraisal

All the articles accepted on full-texts were split into studies – a study corresponding to one species and one method – in order to carry out a critical appraisal and assess their validity. First, a test was conducted on a subsample of studies by two reviewers (RS and AL) before critical appraisal was performed by AL for all studies. To define the criteria of this appraisal, a gold standard protocol was firstly determined in the context of an ideal study, supposedly granted with unlimited financing, time and workforce [37]. Six criteria were identified:

- the number of individuals (Replication criterion),
- the number of measures (Repetition criterion),
- the presence of a control (Control criterion); namely a reference electrode in the case of an electroretinogram or a continuous light for a behavioural experiment,
- the randomisation of individuals throughout experimental groups (Randomisation criterion),
- how specimens were handled before the experiment (Population criterion),
- how specimens were exposed to the treatment during the experiment (Exposition criterion).

Each criterion was assigned a ‘high’, ‘medium’ or ‘low’ risk of bias (see S1 Table for details). Finally, an overall risk of bias was assigned for each accepted study:

- ‘high’ for a study with three high-risk-of-bias criteria,
- ‘medium’ for a study which had a medium risk of bias in the replication or control criteria or three medium-risk-of-bias criteria,
- ‘low’ for remaining studies.

We considered a study to be unreliable, and therefore directly excluded it if there was a total absence of replication or control.

## Data extraction

Several experimental methodologies have been used in the literature to quantify CFF. Electrophysiological protocols are the most frequently used and often involve electroretinograms (ERG) or, alternatively electroencephalograms, which both measure the electrical response of the retina or brain to flickering light. Behavioural protocols have also been used and are mainly of two kinds: either a two-alternative forced choice procedure that requires an animal to choose the preferred light source between the flickering one (usually with increasing frequencies) and the continuous one [28]; or optomotor protocols where the nystagmus reflex of the head or eye of an animal is monitored through increasing flicker frequencies [46].

CFF data were extracted by two reviewers (ML and AL). AL checked all the extracted data by ML, which means that half of the corpus was double checked. Also, a test was conducted on a subsample of studies by two reviewers (RS and AL). As our goal was to determine the maximum CFF at which animals could be impacted, if an article was to test different light intensities, we extracted the maximum CFF recorded by authors. If data were only presented on graphs, we extracted them using WebPlotDigitizer [47]. We also extracted metadata from each study: namely methods, either electrophysiological or behavioural, light sources, light intensities at which CFF were measured. We added a measure of the low, variable or high light exposure levels an animal is likely to experience in natural conditions (following the method from Healy *et al.* (2013) and Inger *et al.* (2014) [33,34]). Briefly, a nocturnal or deep-sea foraging animal is likely to experience a low exposure to light compared to the high exposure of a diurnal or shallow water foraging animal. Cathemeral and crepuscular animals are considered to be exposed to variable quantities of light. Each species was associated with its taxonomic class [48], its trophic level (e.g. primary consumer, omnivorous and predator) and a measure of its body size (i.e. large, average and small). Each study was also assigned its critical appraisal risk of bias.

## Statistical analysis

To assess the influence of methods, light sources, taxonomic classes, light exposure levels, body sizes and trophic levels on CFF, we fitted a linear-mixed effect model (LMM). Using a subset of means, medians, ranges of values, and extrapolated CFF values, we only considered 7 sufficiently represented taxonomic classes, which corresponded to 132 CFF values. Because the number of reported CFF values can vary between species, species was used as a random factor. CFF was square root-transformed to improve normality of residuals. The most parsimonious model was selected and comprised the two fixed factors: taxonomic classes and light exposure levels. To assess to what extent variations of CFF were explained by our model, we computed R_GLMM(m)_^2^, the marginal R^2^ representing the variance explained by the fixed effects and R_GLMM(c)_^2^, the conditional R^2^ representing the variance explained by both fixed and random effects. We also verified the influence of critical appraisal risks of bias on CFF. All statistical analyses were carried out on R software (version 4.1.2). The LMM was computed with the package ‘lmerTest’ [49] and R² thanks to ‘MuMIn’ [50]. Graphs were customized thanks to the ‘ggplot2’ package [51].

# **Results**

## Screening process

The complete literature screening process is presented on Fig 1. All citations and their inclusion/exclusion on titles, abstracts and full-texts, associated with the reason in case of exclusion, are listed in S4 File and S5 File.

At first, 4881 citations were exported from the three databases and 2277 were selected on titles, among which 1042 had an available abstract. Abstracts screening lead to 477 accepted citations. Among them, 445 full-texts PDF were retrieved, of which 106 were kept after the full-text screening step. Over the whole screening process, 45 citations were identified as duplicates and were removed. Sixty-eight additional records came from our call for grey literature but none passed the full-text screening. Lastly, 46 other articles found by the review team that directly dealt with CFF [28,33,34,42–45], but not directly found in the three considered databases, were added to the final corpus.

Overall, 152 articles were included and were split into 267 studies. After the exclusion of studies without any replication nor control, 200 studies were retained. The critical appraisal step resulted in 8 % (16) of studies with a high risk of bias, 78 % (155) with a medium risk of bias and 14 % (29) with a low risk of bias (S6 File).

**Fig 1. ROSES flow diagram reporting the screening process of the articles and studies of the review [52]**

## Bibliometric results

Accepted articles were published between 1935 and 2020 with a major increase in the mid-1990s with less than 10 articles being published every five years before 1990 to more than 40 between 2015 and 2020 (S1 Fig). We classified studies’ methodology into two groups: Electrophysiology (153) and Behavioural (47). In the first group, electroretinogram was the most frequently used methodology even if electroencephalograms and other physiological protocols were also used. For the second group, two-force choice procedures were more often chosen, followed by optomotor ones. In both types of studies, authors used several types of light sources to produce their stimuli, LED being the most frequently chosen one (79) along with gas discharge lamps (37) and monochromators (33). More rarely, incandescent lamps and monitor screens were also used.

Fifteen taxonomic classes were found. Actinopterygii was the most represented taxon with more than 44 species being examined, followed by Malacostraca, Mammalia and Insecta with approximately 30 species (S2 Fig). Aves were studied in 23 species and Elasmobranchii and Reptilia were less examined both with 14 species. Only three Cephalaspidomorphi species were studied and, for each of the 7 remaining taxonomic classes, only one study was carried out. Overall, on the 200 CFF values that were observed, 156 different species were recorded. *Mus musculus* L. was the most examined species with 8 occurrences, along with *Gallus gallus* L. (6 studies) and *Rattus norvegicus* B. (6 studies). The vast majority of species (133) were studied only once.

## Variability of CFF values

CFF was more often reported as a mean value (125 studies) but authors also used the range of values, the minimum or maximum, the median value or the extrapolation from their data. Among the 125 mean values, 77 were detailed by a measure of variation, either a standard error (65) or a standard deviation.

Reported values of CFF varied from a maximum of between 300 Hz and 500 Hz for the beetle *Melanophila acuminata* D. [53] to (0.57 ± 0.08) Hz for the snail *Lissachatina fulica* B. [54]. As the 50 Hz electrical supply in Europe can produce a flickering effect at a frequency of 100 Hz, we considered this threshold to estimate a species potential perception of ALAN’s flicker. When only considering species living under low and variable light exposure levels, seven insects and two fish species among three taxonomic classes, namely Insecta, Aves, and Actinopterygii, had CFF higher than this threshold (Fig 2).

**Fig 2.** **Distribution of maximum Critical Fusion Frequencies (CFF).** Only species living under low and variable light exposure levels in Actinopterygii, Aves, and Insecta, the three classes that had species with CFF higher than 100 Hz, have been represented. The dashed line represents the flicker frequency of a lamp on a 50 Hz electrical supply – i.e. 100 Hz.

## Effects of exposure levels and taxonomy on CFF

To fit our LMM, we used a subset of homogenous CFF values and 7 well represented taxonomic classes, namely Insecta, Aves, Actinopterygii, Elasmobranchii, Malacostraca, Mammalia, and Reptilia, which accounted for 104 species and 132 comparable CFF values. All methods, light sources, light exposure levels, body sizes and trophic levels were well distributed in this subset. The most parsimonious model only comprised the two fixed effects light exposure levels and taxonomic classes, which were both highly significant (F-value = 8.69, df = 2, p-value = 0,0003; F-value = 23.99, df = 6, p-value < 0,0001). Those two fixed effects largely explained the variations of CFF in our model (R_GLMM(m)_^2^ = 0.70; R_GLMM(c)_^2^ = 0.90). We did not find any significant effect of critical appraisal risks of bias on CFF. Based on the distribution of CFF between trophic levels and even though no significant effect was found by our model, we still suspected its potential influence (Fig 3A) and thus carried out an additional analysis, using a random forest - R package ‘randomForest’ [55] - to determine the most important variables which predicted CFF. We found taxonomic classes, trophic and light exposure levels to be the three most important variables, corroborating the results from our LMM and confirming our assumption on trophic levels.

Animals exposed to variable and high levels of light had significantly higher CFF (p-value < 0.001) with a mean (± SD) of 103.4 (± 57.0) Hz and 92.7 (± 71.2) Hz compared to (34.5 ± 23.3) Hz for animals exposed to low light levels (Fig 3B). Over the 7 taxonomic classes of the subset, Insecta had significantly higher CFF than Aves (p-value < 0.001), with respectively (175.2 ± 80.7) Hz and (94.0 ± 28.0) Hz. Both classes had also significantly greater CFF than the other five taxa, which CFF ranged from (57.4 ± 20.2) Hz in Reptilia to (26.6 ± 12.5) Hz in Elasmobranchii (Fig 4, Table 1). Additionally, primary consumer had higher mean CFF with (146.2 ± 73.6) Hz compared to (44.0 ± 29.8) Hz in omnivorous and (48.0 ± 28.1) Hz in predator species.

**Fig 3.** **Square root Critical Fusion Frequency (CFF) for (A) primary consumer, omnivorous and predator trophic levels and for (B) low, variable and high light exposure levels.** The dashed line represents the flicker frequency of a lamp on a 50 Hz electrical supply. Sample size: Primary consumer (n = 20), Omnivorous (n = 62), Predator (n = 47), Low (n = 66), Variable (n = 9), High (n = 57). Linear mixed effect model differences are indicated as follows: *** p-value < 0.001, ** p-value < 0.01, * p-value < 0.05, ns non significant.

**Table 1.** **Linear-mixed models (LMM) results testing for differences of Critical Fusion Frequencies (CFF) between the most studied taxonomic classes.**

|  | Insecta | | Aves | | Reptilia | | Actinopterygii | | Mammalia | | Elasmobranchii | | Malacostraca | |
| --- | --- | --- | --- | --- | --- | --- | --- | --- | --- | --- | --- | --- | --- | --- |
|  | p-value |  | p-value |  | p-value |  | p-value |  | p-value |  | p-value |  | p-value |  |
| Insecta | - |  | 1.29E-05 | *** | 5.13E-08 | *** | 2.44E-18 | *** | 5.45E-09 | *** | 1.22E-14 | *** | 1.41E-14 | *** |
| Aves |  |  | - |  | 0.044 | * | 9.18E-05 | *** | 0.016 | * | 5.04E-05 | *** | 1.33E-04 | *** |
| Reptilia |  |  |  |  | - |  | 0.365 |  | 0.744 |  | 0.073 |  | 0.143 |  |
| Actinopterygii |  |  |  |  |  |  | - |  | 0.596 |  | 0.159 |  | 0.355 |  |
| Mammalia |  |  |  |  |  |  |  |  | - |  | 0.117 |  | 0.223 |  |
| Elasmobranchii |  |  |  |  |  |  |  |  |  |  | - |  | 0.557 |  |
| Malacostraca |  |  |  |  |  |  |  |  |  |  |  |  | - |  |

Significant differences are indicated as follows: *** p-value < 0.001, ** p-value < 0.01, * p-value < 0.05

**Fig 4. Distribution of square root critical fusion frequencies (CFF) across the most studied taxonomic classes.** The dashed line represents the flicker frequency of a lamp on a 50 Hz electrical supply. Sample size: Insecta (n = 18), Aves (n = 15), Reptilia (n = 6), Actinopterygii (n = 34), Mammalia (n = 18), Malacostraca (n = 29), Elasmobranchii (n = 12). Linear mixed effect model significant differences are indicated as follows: *** p-value < 0.001, ** p-value < 0.01, * p-value < 0.05, ns non significant. Least significant differences between classes were chosen to represent significance levels.

# **Discussion**

Before the potential effects of flickering light on species can be assessed, an essential prerequisite is to build a comprehensive knowledge base on CFF values, as it is a measure of the perception of flicker by animals. We thus aimed at updating previous reviews on CFF [33,34] by following the more comprehensive and transparent method of systematic reviews, as preconized by the CEE [37]. We identified that wide gaps of knowledge in CFF values remain to date, for numerous taxa (e.g. Amphibia, Arachnida and some aquatic taxa) and should be filled by further experimental investigations.

When comparing our CFF values to those found by Inger *et al.* (2014) [33], the maximum CFF remained the same - i.e. between 300 Hz and 500 Hz for the beetle *Melanophila acuminata* D. - while a lower minimum was found - i.e. (0.57 ± 0.08) Hz for the snail *Lissachatina fulica* B. - which can be explained by the more extensive literature search strategy carried out here. Even though Hammer *et al.* (2001) [53] acknowledged that their measure of CFF from *M. acuminata*’s pit organ was not accurate, those two upper and lower boundaries for CFF have not been challenged for nearly 20 years. Thus, we can hypothesise that they represent solid limit frequencies within the animal kingdom.

When fitting a similar model as the one by Inger *et al.* (2014) [33] on our broader set of species, the similar overall results were observed in the present study: neither the methodology, electrophysiological or behavioural, nor the light source significantly explained differences in CFF, while taxonomic classes and light exposure levels did. Insecta had the greatest CFF followed by Aves, both of which had significantly higher CFF than the ones observed for all other classes. The latter finding is interesting when considering the current decline of insect populations [56,57] of which ALAN, alongside the loss of natural habitats and the use of pesticides [58], may be one of the main drivers [4,5]. We can, therefore, hypothesise that flicker might represent a meaningful component of light pollution, along with intensity, spectral composition and temporality.

Even though we were not able to show any significant effect of trophic levels with our model, a random forest regression showed that trophic levels were, indeed, an important variable in predicting CFF values. In fact, this relationship between prey and predator have already been discussed extensively. Potier *et al.* (2019) [28] have linked the temporal resolution, defined as the ability to resolve rapid movements and estimated by the CFF, to the speed of the prey of diurnal raptors, and showed that the peregrine falcon, which hunts birds, had a higher temporal resolution than the Harris’s hawk which eats mainly terrestrial mammals. Likewise, in marine habitats, McComb *et al.* (2010) [30] related the higher temporal resolutions of the bonnethead and scalloped hammerhead sharks to their shallow and bright reef habitats and their fast-moving prey compared to the lower temporal resolution of the blacknose shark, which usually forages in deeper and dimmer water environments. Differences in temporal resolution may even rely on a co-evolutionary relationship between the prey and the predator. Indeed, Boström *et al.* (2016) [29] argued that the high speed of insect prey could represent a major driver of high temporal resolutions in flycatchers, their bird predator, which could, then, drive those of insects further away. Our data showed that primary consumer may have greater CFF than species on higher levels of the food chain, which could mean that prey have a head start on temporal resolution abilities. Such assumptions should, nevertheless, be studied more thoroughly in the future.

Similar to our examination of trophic levels, our model did not show any significant effect of body size on CFF. However, one could think that the smaller the animal, the higher the CFF might be (S3 Fig). Our analysis also showed that nocturnal animals had significantly lower CFF than crepuscular and diurnal ones, which is in line with the often reported trade-off between light sensitivity and temporal resolution – i.e. higher light levels are needed for high rates of sampling [30,33,34]. Those two previous results have already been assessed by Healy *et al.* (2013) [34] who demonstrated the correlation between body mass, metabolic rate and temporal resolution – i.e. small animals with high metabolic rates in high light environments tend to have much higher CFF than large animals with low metabolic rate in darker environments.

## The perception of artificial anthropogenic light flicker

Due to the alternating nature of power supply, some anthropogenic light sources may flicker at a frequency of 100 Hz. This flicker have been thoroughly studied in fluorescent lighting [24,33,59,60] but can be found in vapour discharge luminaires as well. When the latter are still used for outdoor lighting, it has hardly ever been the case for fluorescent lighting. Be that as it may, both types are increasingly being replaced by LEDs, that are proving much more energy-efficient. Various LED technologies have been engineered and each one may or may not show a 100-Hz flickering effect depending on the quality of their driver, the electronic component supplying power to the diode, and their dimming technology which relies, in the case of Pulse Width Modulation (PWM) technology, on rapid ON/OFF sequences at varying frequencies (usually 100 Hz– 400 Hz) according to the chosen light intensity.

We thus considered the 100-Hz threshold to be relevant in order to assess the potential perception of anthropogenic flicker by animals. By isolating nocturnal and crepuscular animals, we were able to identify seven insects and two fish species which were much more likely to experience adverse effects from artificial light flicker. As we observed important intra-specific variation in CFF, we could also expect that some other species near the 100-Hz threshold are at risk. Chatterjee *et al.*, (2020) [61] provided[?] the latest data on nocturnal and crepuscular insects CFF and looking at maximum values, they measured CFF of 230 Hz, 173 Hz and 149 Hz, respectively in the crepuscular butterflies *Melanitis leda* L., *Gangara thyrsis* F., *Hasora chromus* C., and 146 Hz, 116 Hz, 110 Hz in the nocturnal butterflies *Hippotion* sp. H., *Daphnis nerii* L., and *Eupatula macrops* L., finally covering one of the poorly studied groups that Inger *et al.* (2014) [33] highlighted. Contrary to Inger *et al.* (2014) [33] who argued that the actual perception of flicker by animals should be limited as they found that only diurnal animals were able to perceive flicker, we identified some crepuscular and nocturnal species that could, in fact, be influenced by flickering ALAN. Nonetheless, as Inger *et al.* (2014) [33] also stated, many animals may not be able to perceive flicker as CFF diminishes with light intensity and therefore distance to the light source.

For as crucial assessing an animal actual perception of flicker is, it has to be noted that animals could still be subjected to the deleterious effects of a theoretically consciously unperceivable flicker (i.e. a flicker that an animal would not be able to perceive based on its CFF), thus challenging the wider use of CFF to measure the impacts of flicker on species. Indeed, Lu *et al.* (2012) [62] argued that a chromatic flicker at frequencies between 42.5 and 75 Hz, superior to the human CFF and consciously unperceivable, was still able to entail their human subjects’ alerting and orienting attentional networks.

## Knowledge gaps

As it was first noted by Inger *et al.* (2014) [33] and even if more and more studies have been carried out lately, an important lack of data on animals’ CFF remains and is hampering our understanding of the impacts of anthropogenic lighting on biodiversity. We, thence, reiterate the important need of additional primary research in order to improve our knowledge of CFF in as many species as possible. Indeed, on the millions of species that populate the Earth, just over 150 were studied thus far accounting for only 15 taxonomic classes. This scarcity of data is particularly noticeable in key nocturnal taxa, which are likely to be greatly disturbed by ALAN. As such, the flicker perception of many more nocturnal and crepuscular insect species should be studied as well as those of nocturnal birds and bats, two other groups on which no additional data was retrieved in our systematic review compared to previous reviews [33,34].

Additionally, based on critical appraisal, we identified that very few studies achieved a low risk of bias according to the criteria defined in this review. For instance, a large number of authors failed to indicate if they had performed any repetition or randomisation. Likewise, very little information was provided on the state of animals prior to the experiments in some studies. Too few studies in our corpus used several (or not as many as needed) light intensities in order to reach the true maximum flicker fusion frequency that is the CFF. Some studies could not report true maximum FFF as the highest frequency that their apparatuses could attain was too low for their specimens, resulting in minimum CFF values in our corpus which may not be informative. We also advocate the need for a strong and consistent reporting of CFF data. Indeed, accurate values of CFF should absolutely be reported as a mean attached to a measure of variation, which only one third of our studies provided, despite it is a basic statistical need. Last but not least, we remind the essential need for replications and controls in order to measure reliable CFF values. There, nevertheless, remains many recent studies using only one specimen or failing to report the use of a reference electrode for electroretinograms that had to be excluded after critical appraisal. Even if we fully understand the difficulties to collect and/or train a high number of individuals, it is nonetheless fundamental to provide more than a unique individual value to ensure the relevance of a species threshold of flicker perception.

## Review limitations

Due to time limitation and financing constraints, we sometimes had to decide to reduce our requirements comparing to CEE guidelines. First, we could not include search engines (e.g. Google Scholar) in our search strategy, nor could we request supplementary databases (e.g. Scopus) which would have probably increased the reliability of our test list by increasing the number of test list articles indexed in the requested databases. In addition, after title screening, we had to set aside citations that did not possess an appended abstract. Indeed, dealing with these citations would have meant searching for the full-text of these 1235 citations, which represented an additional workload unfeasible within the scope of our project. However, this additional work remains feasible subsequently thanks to the S7 File which lists these citations without abstract. We also could not exploit the reviews (i.e. articles that might include some CFF values through their full-text) identified through citation screening -except Inger *et al.* (2014) [33] and Healy *et al.* (2013) [34] - and we then collated them in S8 File for further exploitation. We are conscious that these shortcomings may lead to an underestimation of the number of identified CFF, which could potentially have been assessed in more species, and could provide less robust data as more values for a given species might have been found. Nevertheless, our database already provides many additional CFF values compared to previously published reviews [33,34].

Lastly, studies were only critically appraised by one reviewer whereas the CEE guidelines call for a double independent assessment by two reviewers. However, we performed a test between two reviewers on a subsample of articles before starting critical appraisal. Accordingly, we could not carry out a double independent data extraction on the whole corpus, as requested by the CEE, and only performed the double independent extraction on one half of the corpus.

# **Conclusions**

This systematic review, based on the method preconized by the CEE, provided a database of 200 critical fusion frequency values for 156 different species. This database complements the existing reviews on CFF by providing a wider range of more confident values. It, then, represents a current and qualitative state of knowledge of flicker perception in the animal kingdom, which could be helpful for scientists and researchers as well as for practitioners, such as lighting managers and designers. Here, we found that the beetle *Melanophila acuminata* D. could perceive the highest flicker frequency among all the recorded species whereas the snail *Lissachatina fulica* B. had the lowest temporal resolution. Insects and birds were the two taxa with the highest CFF. Moreover, nocturnal species had lower CFF than diurnal and crepuscular ones. We observed that some nocturnal and crepuscular insects could potentially perceive the flicker of anthropogenic light sources, which means that they could be subjected to its deleterious adverse effects. We also showed that trophic levels may be an important variable in predicting CFF. Nevertheless, the number of collected CFF values remains very low in relation to the number of all living animal species. In addition, this lack of data especially concerns key nocturnal taxa like birds and bats that are likely to be exposed to outdoor lightings. Then, this result is necessarily provisional. We thus argue that many more species should be pressingly investigated by researchers to improve our knowledge of flicker perception by animals.

# **Acknowledgments**

We would like to thank Simon Potier who enlightened us about behavioural and optomotor CFF discrimination protocols. We also wish to thank Françoise Viénot, Christophe Martinsons, Jack Falcón, Christian Kerbiriou, Léa Mariton and Matthieu Iodice for providing us with literature, as well as Dakis-Yaoba Ouédraogo for her statistical advice which contributed to substantially improve the quality of our manuscript. At last, we sincerely thank Marie-Pierre Alexandre for her support and advice all along our study.

# **References**

1.	Intergovernmental Science-Policy Platform on Biodiversity and Ecosystem Services. Summary for policymakers of the global assessment report on biodiversity and ecosystem services of the Intergovernmental Science-Policy Platform on Biodiversity and Ecosystem Services. 2019.

2.	International Union for Conservation of Nature. UICN Red List 2017-2020 Report. 2021. Available from: https://nc.iucnredlist.org/redlist/resources/files/1630480997-IUCN_RED_LIST_QUADRENNIAL_REPORT_2017-2020.pdf

3.	Sánchez de Miguel A, Bennie J, Rosenfeld E, Dzurjak S, Gaston KJ. First Estimation of Global Trends in Nocturnal Power Emissions Reveals Acceleration of Light Pollution. Remote Sensing. 2021;13: 3311. doi:10.3390/rs13163311

4.	Hölker F, Wolter C, Perkin EK, Tockner K. Light pollution as a biodiversity threat. Trends Ecol Evol. 2010;25: 681–682. doi:10.1016/j.tree.2010.09.007

5.	Hölker F, Bolliger J, Davies TW, Giavi S, Jechow A, Kalinkat G, et al. 11 Pressing Research Questions on How Light Pollution Affects Biodiversity. Front Ecol Evol. 2021;9: 767177. doi:10.3389/fevo.2021.767177

6.	Davies TW, Smyth T. Why artificial light at night should be a focus for global change research in the 21st century. Glob Change Biol. 2018;24: 872–882. doi:10.1111/gcb.13927

7.	Falcón J, Torriglia A, Attia D, Viénot F, Gronfier C, Behar-Cohen F, et al. Exposure to Artificial Light at Night and the Consequences for Flora, Fauna, and Ecosystems. Front Neurosci. 2020;14: 602796. doi:10.3389/fnins.2020.602796

8.	Pérez Vega C, Zielinska-Dabkowska KM, Schroer S, Jechow A, Hölker F. A Systematic Review for Establishing Relevant Environmental Parameters for Urban Lighting: Translating Research into Practice. Sustainability. 2022;14: 1107. doi:10.3390/su14031107

9.	ffrench-Constant RH, Somers-Yeates R, Bennie J, Economou T, Hodgson D, Spalding A, et al. Light pollution is associated with earlier tree budburst across the United Kingdom. Proc R Soc B. 2016;283: 20160813. doi:10.1098/rspb.2016.0813

10.	Owens ACS, Cochard P, Durrant J, Farnworth B, Perkin EK, Seymoure B. Light pollution is a driver of insect declines. Biological Conservation. 2020;241: 108259. doi:10.1016/j.biocon.2019.108259

11.	Dickerson AL, Hall ML, Jones TM. The effect of natural and artificial light at night on nocturnal song in the diurnal willie wagtail. Science of The Total Environment. 2022;808: 151986. doi:10.1016/j.scitotenv.2021.151986

12.	Fobert EK, Burke da Silva K, Swearer SE. Artificial light at night causes reproductive failure in clownfish. Biol Lett. 2019;15: 20190272. doi:10.1098/rsbl.2019.0272

13.	Knop E, Zoller L, Ryser R, Gerpe C, Hörler M, Fontaine C. Artificial light at night as a new threat to pollination. Nature. 2017;548: 206–209. doi:10.1038/nature23288

14.	Lewanzik D, Voigt CC. Artificial light puts ecosystem services of frugivorous bats at risk. Pocock M, editor. J Appl Ecol. 2014;51: 388–394. doi:10.1111/1365-2664.12206

15.	Gaston KJ, Bennie J, Davies TW, Hopkins J. The ecological impacts of nighttime light pollution: a mechanistic appraisal: Nighttime light pollution. Biol Rev. 2013;88: 912–927. doi:10.1111/brv.12036

16.	Simons AL, Martin KLM, Longcore T. Determining the Effects of Artificial Light at Night on the Distributions of Western Snowy Plovers (Charadrius nivosus nivosus) and California Grunion (Leuresthes tenuis) in Southern California. Journal of Coastal Research. 2021;38. doi:10.2112/JCOASTRES-D-21-00107.1

17.	Secondi J, Mondy N, Gippet JMW, Touzot M, Gardette V, Guillard L, et al. Artificial light at night alters activity, body mass, and corticosterone level in a tropical anuran. Behavioral Ecology. 2021;32: 932–940. doi:10.1093/beheco/arab044

18.	Diamantopoulou C, Christoforou E, Dominoni DM, Kaiserli E, Czyzewski J, Mirzai N, et al. Wavelength-dependent effects of artificial light at night on phytoplankton growth and community structure. Proc R Soc B. 2021;288: 20210525. doi:10.1098/rspb.2021.0525

19.	Kühne JL, van Grunsven RHA, Jechow A, Hölker F. Impact of Different Wavelengths of Artificial Light at Night on Phototaxis in Aquatic Insects. Integr Org Biol. 2021;61: 1182–1190. doi:10.1093/icb/icab149

20.	Spoelstra K, van Grunsven RHA, Ramakers JJC, Ferguson KB, Raap T, Donners M, et al. Response of bats to light with different spectra: light-shy and agile bat presence is affected by white and green, but not red light. Proc R Soc B. 2017;284: 20170075. doi:10.1098/rspb.2017.0075

21.	Azam C, Kerbiriou C, Vernet A, Julien J-F, Bas Y, Plichard L, et al. Is part-night lighting an effective measure to limit the impacts of artificial lighting on bats? Glob Change Biol. 2015;21: 4333–4341. doi:10.1111/gcb.13036

22.	Day J, Baker J, Schofield H, Mathews F, Gaston KJ. Part-night lighting: implications for bat conservation: Part-night lighting and bats. Anim Conserv. 2015;18: 512–516. doi:10.1111/acv.12200

23.	International Commission on Non-Ionizing Radiation Protection. Light-Emitting Diodes (LEDS): Implications for Safety. Health Physics. 2020;118: 549–561. doi:10.1097/HP.0000000000001259

24.	Greenwood VJ, Smith EL, Goldsmith AR, Cuthill IC, Crisp LH, Walter-Swan MB, et al. Does the flicker frequency of fluorescent lighting affect the welfare of captive European starlings? Appl Anim Behav Sci. 2004;86: 145–159. doi:10.1016/j.applanim.2003.11.008

25.	Sautter CS, Cocchi L, Schenk F. Dynamic visual information plays a critical role for spatial navigation in water but not on solid ground. Behavioural Brain Research. 2008;194: 242–245. doi:10.1016/j.bbr.2008.07.006

26.	Barroso A, Haifig I, Janei V, da Silva I, Dietrich C, Costa-Leonardo A. Effects of flickering light on the attraction of nocturnal insects. Lighting Res Technol. 2017;49: 100–110. doi:10.1177/1477153515602143

27.	Wilkins AJ, Nimmo-Smith I, Slater AI, Bedocs L. Fluorescent lighting, headaches and eyestrain. Lighting Res Technol. 1989;21: 11–18. doi:10.1177/096032718902100102

28.	Potier S, Lieuvin M, Pfaff M, Kelber A. How fast can raptors see? Journal of Experimental Biology. 2019; jeb.209031. doi:10.1242/jeb.209031

29.	Boström JE, Dimitrova M, Canton C, Håstad O, Qvarnström A, Ödeen A. Ultra-Rapid Vision in Birds. PLoS ONE. 2016;11: e0151099. doi:10.1371/journal.pone.0151099

30.	McComb DM, Frank TM, Hueter RE, Kajiura SM. Temporal Resolution and Spectral Sensitivity of the Visual System of Three Coastal Shark Species from Different Light Environments. Physiol Biochem Zool. 2010;83: 299–307. doi:10.1086/648394

31.	Boström JE, Haller NK, Dimitrova M, Ödeen A, Kelber A. The flicker fusion frequency of budgerigars (Melopsittacus undulatus) revisited. J Comp Physiol A. 2017;203: 15–22. doi:10.1007/s00359-016-1130-z

32.	Frank TM. Comparative Study of Temporal Resolution in the Visual Systems of Mesopelagic Crustaceans. The Biological Bulletin. 1999;196: 137–144. doi:10.2307/1542559

33.	Inger R, Bennie J, Davies TW, Gaston KJ. Potential Biological and Ecological Effects of Flickering Artificial Light. Boyles JG, editor. PLoS ONE. 2014;9: e98631. doi:10.1371/journal.pone.0098631

34.	Healy K, McNally L, Ruxton GD, Cooper N, Jackson AL. Metabolic rate and body size are linked with perception of temporal information. Animal Behaviour. 2013;86: 685–696. doi:10.1016/j.anbehav.2013.06.018

35.	Sutherland WJ, Pullin AS, Dolman PM, Knight TM. The need for evidence-based conservation. Trends Ecol Evol. 2004;19: 305–308. doi:10.1016/j.tree.2004.03.018

36.	Walsh JC, Dicks LV, Sutherland WJ. The effect of scientific evidence on conservation practitioners’ management decisions. Conservation Biology. 2015;29: 88–98. doi:10.1111/cobi.12370

37.	Collaboration for Environmental Evidence. Guidelines and standards for evidence synthesis in environmental management. Version 5.0. In: Pullin AS, Frampton GK, Livoreil B, Petrokofsky G, editors. 2018 [cited 2022 Apr 7]. Available from: http://www.environmentalevidence.org/information-for-authors.

38.	Berger-Tal O, Greggor AL, Macura B, Adams CA, Blumenthal A, Bouskila A, et al. Systematic reviews and maps as tools for applying behavioral ecology to management and policy. Behavioral Ecology. 2019;30: 1–8. doi:10.1093/beheco/ary130

39.	Haddaway NR, Bernes C, Jonsson B-G, Hedlund K. The benefits of systematic mapping to evidence-based environmental management. Ambio. 2016;45: 613–620. doi:10.1007/s13280-016-0773-x

40.	Dicks LV, Walsh JC, Sutherland WJ. Organising evidence for environmental management decisions: a ‘4S’ hierarchy. Trends Ecol Evol. 2014;29: 607–613. doi:10.1016/j.tree.2014.09.004

41.	Haddaway N, Macura B, Whaley P, Pullin A. ROSES for Systematic Map Reports. Version 1.0. 2018. doi:10.6084/m9.figshare.5897299

42.	Lisney TJ, Rubene D, Rózsa J, Løvlie H, Håstad O, Ödeen A. Behavioural assessment of flicker fusion frequency in chicken Gallus gallus domesticus. Vision Research. 2011;51: 1324–1332. doi:10.1016/j.visres.2011.04.009

43.	Ryan LA, Hemmi JM, Collin SP, Hart NS. Electrophysiological measures of temporal resolution, contrast sensitivity and spatial resolving power in sharks. J Comp Physiol A. 2017;203: 197–210. doi:10.1007/s00359-017-1154-z

44.	Warrington RE, Hart NS, Potter IC, Collin SP, Hemmi JM. Retinal temporal resolution and contrast sensitivity in the parasitic lamprey Mordacia mordax and its non-parasitic derivative M . praecox. Journal of Experimental Biology. 2017; jeb.150383. doi:10.1242/jeb.150383

45.	Woo KL, Hunt M, Harper D, Nelson NJ, Daugherty CH, Bell BD. Discrimination of flicker frequency rates in the reptile tuatara (Sphenodon). Naturwissenschaften. 2009;96: 415–419. doi:10.1007/s00114-008-0491-8

46.	Jenssen TA, Swenson B. An ecological correlate of critical flicker-fusion frequencies for some Anolis lizards. Vision Research. 1974;14: 965–970. doi:10.1016/0042-6989(74)90164-3

47.	Rohatgi A. WebPlotDigitizer. Version 4.3. 2020 [cited 2022 Apr 7]. Available from: https://automeris.io/WebPlotDigitizer

48.	GBIF.org [Internet]. Global Biodiversity Information Facility Secretariat: GBIF Backbone Taxonomy. 2021 [cited 2021 Dec 13]. Available from: https://doi.org/10.15468/39omei

49.	Kuznetsova A, Brockhoff PB, Christensen RHB. lmerTest Package: Tests in Linear Mixed Effects Models. J Stat Soft. 2017;82. doi:10.18637/jss.v082.i13

50.	Barton K. Model selection and model averaging based on information criteria (AICc and alike). Version 1.46.0. 2022 [cited 2022 Apr 7]. Available from: http://cran.rproject.org/web/packages/MuMIn/index.html

51.	Wickham H. ggplot2: Elegant Graphics for Data Analysis. 2nd ed. 2016. Cham: Springer International Publishing : Imprint: Springer; 2016. doi:10.1007/978-3-319-24277-4

52.	Haddaway N, Macura B, Whaley P, Pullin A. ROSES flow diagram for systematic maps. Version 1.0. 2018. doi:10.6084/m9.figshare.6085940

53.	Hammer DX, Schmitz H, Schmitz A, Grady H, Iii R, Welch AJ. Sensitivity threshold and response characteristics of infrared detection in the beetle Melanophila acuminata (Coleoptera: Buprestidae). Comp Biochem Physiol Part A. 2001;128: 805–819. doi:10.1016/s1095-6433(00)00322-6

54.	Bobkova MV, Tartakovskaya OS, Borissenko SL, Zhukov VV, Meyer-Rochow VB. Restoration of morphological and functional integrity in the regenerating eye of the giant African land snail Achatina fulica: Eye regeneration in Achatina fulica. Acta Zoologica. 2004;85: 1–14. doi:10.1111/j.0001-7272.2004.00152.x

55.	Liaw A, Wiener M. Classification and Regression by randomForest. R News. 2002;2: 18–22. Available from: https://CRAN.R-project.org/doc/Rnews/

56.	Sánchez-Bayo F, Wyckhuys KAG. Worldwide decline of the entomofauna: A review of its drivers. Biological Conservation. 2019;232: 8–27. doi:10.1016/j.biocon.2019.01.020

57.	Tallamy DW, Shriver WG. Are declines in insects and insectivorous birds related? Ornithological Applications. 2021;123: duaa059. doi:10.1093/ornithapp/duaa059

58.	Spiller KJ, Dettmers R. Evidence for multiple drivers of aerial insectivore declines in North America. The Condor. 2019;121: duz010. doi:10.1093/condor/duz010

59.	Evans JE, Smith EL, Bennett ATD, Cuthill IC, Buchanan KL. Short-term physiological and behavioural effects of high- versus low-frequency fluorescent light on captive birds. Animal Behaviour. 2012;83: 25–33. doi:10.1016/j.anbehav.2011.10.002

60.	Maddocks SA, Goldsmith AR, Cuthill IC. The Influence of Flicker Rate on Plasma Corticosterone Levels of European Starlings, Sturnus vulgaris. Gen Comp Endocrinol. 2001;124: 315–320. doi:10.1006/gcen.2001.7718

61.	Chatterjee P, Mohan U, Krishnan A, Sane SP. Evolutionary constraints on flicker fusion frequency in Lepidoptera. J Comp Physiol A. 2020;206: 671–681. doi:10.1007/s00359-020-01429-3

62.	Lu S, Cai Y, Shen M, Zhou Y, Han S. Alerting and orienting of attention without visual awareness. Consciousness and Cognition. 2012;21: 928–938. doi:10.1016/j.concog.2012.03.012

**Supporting information**

**S1 File. ROSES reporting standards (excel file)**

**S2 File. PRISMA checklist (PDF file)**

**S3 File. Articles from the test list (excel file)**

**S4 File. Citation screening (excel file)**

**S5 File. Citations excluded at full text (excel file)**

**S6 File. Studies critical appraisal (excel file)**

**S7 File. Citations without an abstract (excel file)**

**S8 File. Reviews identified through screening (excel file)**

**S9 File. Unobtainable articles at full text (excel file)**

**S10 File. CFF dataset (excel file)**

**S1 Table. Critical appraisal criteria and decisions**

**S1 Fig. Number of studies by year of publication**

**S2 Fig. Number of species by studied taxonomic class**

**S3 Fig. Square root Critical Fusion Frequency (CFF) for small, average and large animals.** The dashed line represents the flicker frequency of a lamp on a 50 Hz electrical supply. Sample size: Low (n = 96), Average (n = 19), Large (n = 17).

**S4 Fig. Random forest variable importance plot.**
