# Supplementary material for: A flashing light may not be that flashy: A systematic review on critical fusion frequencies
Source: PLoS One. 2022 Dec 30;17(12):e0279718. doi: 10.1371/journal.pone.0279718 (PMC9803175; doi:10.1371/journal.pone.0279718)
Supplement: Attachment — Submitted filename: Response to Editor.docx [file pone.0279718.s014.docx]

**Rebuttal letter**

***Editor:***

The authors have responded to reviewer comments and made extensive revisions. The manuscript is improved and nearly acceptable. I suggest one additional revision. The formulation of research questions is an improvement. However, the first question, "what evidence exists regarding animal CFF?," does not seem to capture the authors' objectives. That question suggests that CFF might not exist or is somehow controversial. Perhaps the authors can delete this first question, elevate their second question to the first question and add a new second question that connects to the statistical analysis of differences among taxa (Table 1). For example: Are there differences in CFF between major taxonomic lineages? (or something to similar).

=> We are thankful for your acknowledgement of our work in revising the MS. Based on your comment and because we acknowledge this first question might be misleading, we revised our review questions: “what is the distribution of CFF between species? Are there differences in how flicker is perceived between taxonomic classes? Which species are more at risk of being impacted by artificial lighting flicker?”
